# Dichlorido{(2*E*)-2-[phen­yl(pyridin-2-yl)methyl­idene]hydrazinecarbo­thio­amide}cadmium(II) methanol monosolvate

**DOI:** 10.1107/S1600536814015694

**Published:** 2014-07-17

**Authors:** Ambili A. Aravindakshan, V. Seena, M. Sithambaresan, M. R. Prathapachandra Kurup

**Affiliations:** a Department of Applied Chemistry, Cochin University of Science and Technology, Kochi 682 022, India; b Department of Chemistry, Faculty of Science, Eastern University, Sri Lanka, Chenkalady, Sri Lanka

**Keywords:** crystal structure

## Abstract

In the title compound, [CdCl_2_(C_13_H_12_N_4_S)]·CH_3_OH, the coord­ination geometry of the Cd^II^ ion is slightly distorted square-pyramidal, as indicated by the τ index of 0.36 (8). The S atom, two N atoms from the pyridyl-azomethine moiety and one of the Cl atoms comprise the basal plane, while the other Cl atom occupies the apical position. The hydrazinecarbo­thio­amide moiety adopts an *E* conformation with respect to the azomethine bond. The solvate mol­ecule in the crystal lattice plays a major role in inter­connecting adjacent mol­ecules by means of O—H⋯Cl and N—H⋯O hydrogen-bonding inter­actions. A supra­molecular three-dimensional architecture is sustained in terms of further N—H⋯Cl and C—H⋯Cl hydrogen-bonding inter­actions.

## Related literature   

For metal complexes of hydrazinecarbo­thio­amide and its derivatives, see: Sreekanth *et al.* (2004 ▶). For applications of hydrazinecarbo­thio­amides, see: Joseph *et al.* (2004 ▶); Kumar *et al.* (2011 ▶, 2013 ▶). For the synthesis of related compounds, see: Philip *et al.* (2006 ▶). For related structures, see: Kunnath *et al.* (2012 ▶). For the calculation of the τ index, see: Addison *et al.* (1984 ▶).
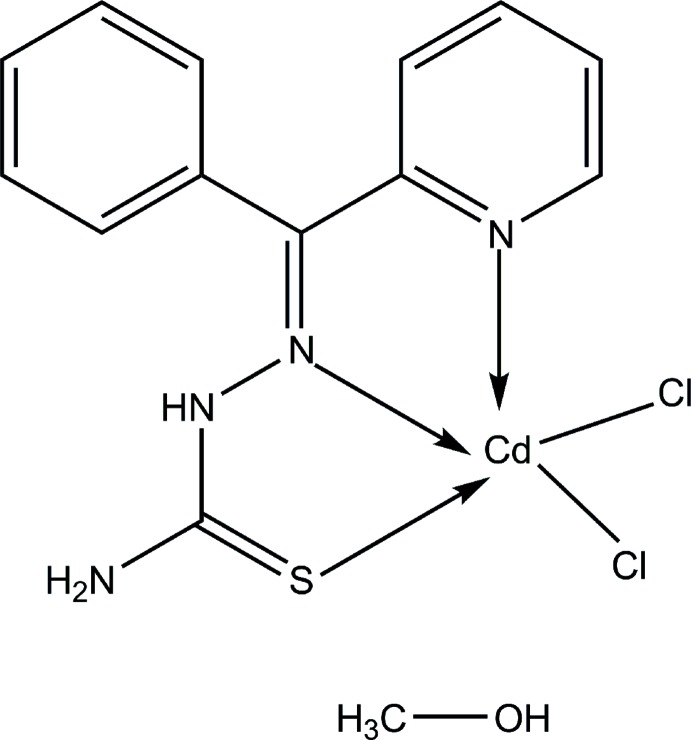


## Experimental   

### 

#### Crystal data  

[CdCl_2_(C_13_H_12_N_4_S)]·CH_4_O
*M* *_r_* = 471.67
Monoclinic, 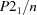

*a* = 7.7213 (4) Å
*b* = 12.9759 (8) Å
*c* = 18.3318 (11) Å
β = 95.248 (2)°
*V* = 1828.98 (18) Å^3^
*Z* = 4
Mo *K*α radiation
μ = 1.61 mm^−1^
*T* = 293 K
0.30 × 0.25 × 0.20 mm

#### Data collection  

Bruker Kappa APEXII CCD diffractometer
Absorption correction: multi-scan (*SADABS*; Bruker, 2004 ▶) *T*_min_ = 0.624, *T*_max_ = 0.725
13483 measured reflections
4392 independent reflections
3804 reflections with *I* > 2σ(*I*)
*R*_int_ = 0.031

#### Refinement  

*R*[*F* ^2^ > 2σ(*F* ^2^)] = 0.028
*wR*(*F* ^2^) = 0.074
*S* = 0.99
4392 reflections
226 parameters
5 restraints
H atoms treated by a mixture of independent and constrained refinement
Δρ_max_ = 0.50 e Å^−3^
Δρ_min_ = −0.60 e Å^−3^

### 

Data collection: *APEX2* (Bruker, 2004 ▶); cell refinement: *APEX2* and *SAINT* (Bruker, 2004 ▶); data reduction: *SAINT* and *XPREP* (Bruker, 2004 ▶); program(s) used to solve structure: *SHELXS97* (Sheldrick, 2008 ▶); program(s) used to refine structure: *SHELXL2008* (Sheldrick, 2008 ▶); molecular graphics: *ORTEP-3 for Windows* (Farrugia, 2012 ▶) and *DIAMOND* (Brandenburg, 2010 ▶); software used to prepare material for publication: *SHELXL97* (Sheldrick, 2008 ▶) and *publCIF* (Westrip, 2010 ▶).

## Supplementary Material

Crystal structure: contains datablock(s) I, global. DOI: 10.1107/S1600536814015694/fj2677sup1.cif

Structure factors: contains datablock(s) I. DOI: 10.1107/S1600536814015694/fj2677Isup2.hkl

CCDC reference: 1012335

Additional supporting information:  crystallographic information; 3D view; checkCIF report

## Figures and Tables

**Table 1:** Hydrogen-bond geometry (Å, °)

*D*—H⋯*A*	*D*—H	H⋯*A*	*D*⋯*A*	*D*—H⋯*A*
N4—H4*A*⋯O1*S*	0.85 (1)	2.14 (2)	2.890 (3)	148 (2)
N4—H4*B*⋯Cl2^i^	0.84 (1)	2.43 (1)	3.253 (2)	167 (3)
N3—H3′⋯O1*S*	0.88 (1)	2.15 (2)	2.924 (3)	147 (3)
O1*S*—H1′⋯Cl1^ii^	0.85 (1)	2.40 (2)	3.201 (3)	158 (4)
C2—H2⋯Cl1^iii^	0.93	2.80	3.680 (3)	159

Symmetry codes: (i) 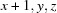; (ii) 
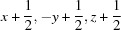
; (iii) 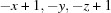.

**Fig. 1.**
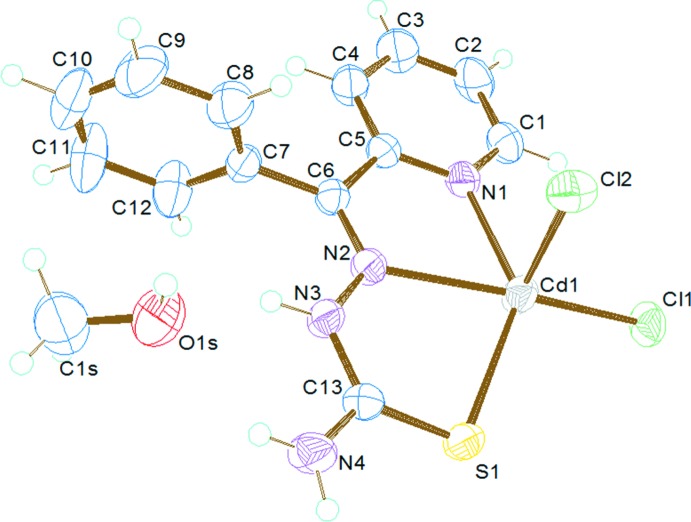
ORTEP view of the compound, drawn with 50% probability displacement ellipsoids for the non-H atoms.

**Fig. 2.**
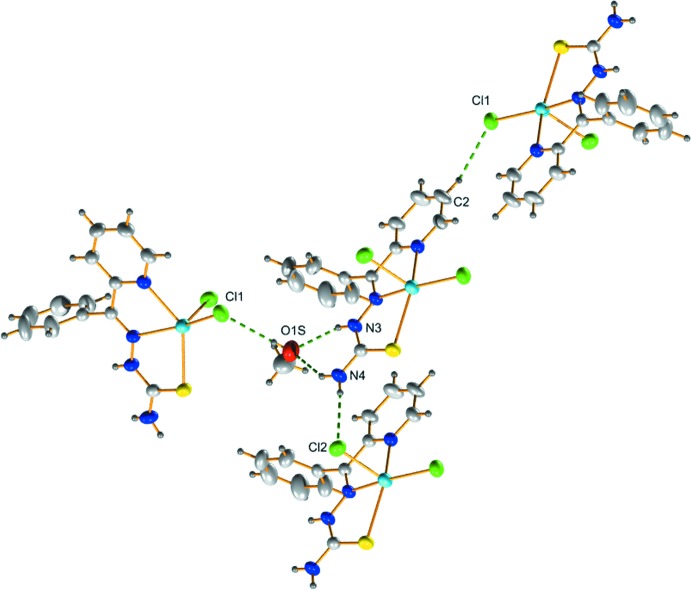
Graphical representation showing hydrogen bonding interactions in the crystal structure of [C13H12CdCl2N4S]·(CH4O).

**Fig. 3.**
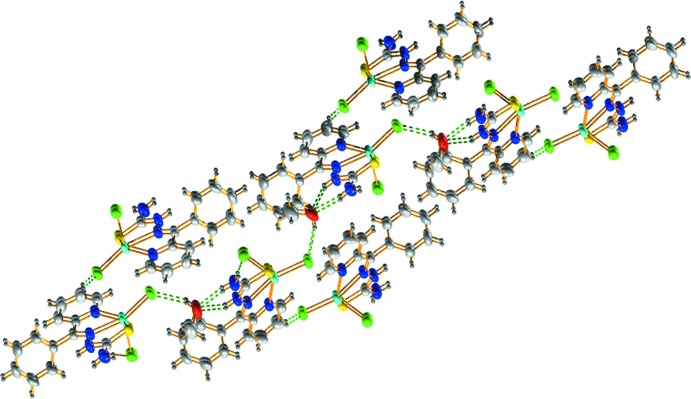
The hydrogen bonding interactions build a double layer progressing along c axis in the title compound.

**Fig. 4.**
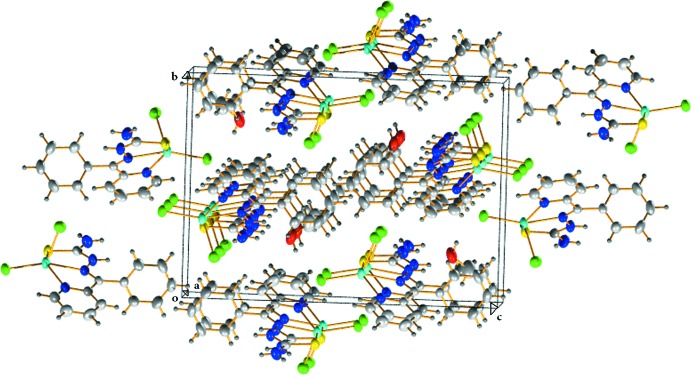
A view of the unit cell along a axis.

## supplementary crystallographic information

### S1. Comment

The importance of hydrazinecarbothioamide is increasing in various fields due to its wide range of medicinal applications (Joseph *et al.*, 2004) and structural diversity due to their variable coordinative abilities (Sreekanth *et al.*, 2004) arising from thioamido-thioiminol tautomerism. Moreover it also found to serve as a corrosion inhibitor on mild steel in HCl (Kumar *et al.*, 2011). Recently a new heterocyclic hydrazinecarbothioamide has been developed as colorimetric and turn on fluorescent sensors for fluoride anion (Kumar *et al.*, 2013).

The title complex [C_13_H_12_CdCl_2_N_4_S]·(CH_4_O) adopts an *E* configuration with respect to C6═N2 bond and the tridentate ligand has its coordinating entities disposed in a *cis* fashion to each other (Fig. 1). The Cd atom in the complex is *N,N^'^,S* chelated by the thioamido form of the hydrazinecarbothioamide ligand. The C6═N2 [1.278 (3) Å] and C13=S1 [1.690 (2) Å] bond distances, very close to the formal C═N and C═S bond lengths respectively confirm the azomethine bond formation and the coordination *via* thioamido form. The coordination geometry around Cd(II) ion is almost square pyramidal (Addison *et al.*, 1984) with a slight distortion (τ = 0.36 (8)). The S1 atom of the hydrazinecarbothioamide moiety, the imino N2 atom, pyridine N1 atom and the Cl1 atom comprise the basal plane while the apical position is occupied by the Cl2 atom (Kunnath *et al.*, 2012). However, the deviation from the ideal square pyramidal geometry is observed by the displacement of Cd atom from the basal plane and the *trans* angle of the basal atoms (Table 1).

There are four classical O–H···Cl, N–H···O and N–H···Cl and one non-classical C–H···Cl intermolecular hydrogen bonding interactions (Table 1, Fig. 2). Three of the classical interactions connect two neighbouring complexes through a solvate molecule with D···A distances of 3.201 (3), 2.924 (3) and 2.890 (3) Å. The other two classical and non-classical interactions directly connect two more neighbouring molecules directly to the main molecule with D···A distances of 3.253 (2) and 3.680 (3) Å respectively. These hydrogen bonding interactions build a double layer (Fig. 3) supramolecular chain along *c* axis. In addition to this, there are two very weak π···π interactions present with *Cg*···*Cg* distances of greater than 4 Å. Fig. 4 shows the packing diagram of the title compound along *a* axis.

### S2. Experimental

The potentialy tridentate ligand (2E)-2-[phenyl(pyridin-2-yl)methylidene]hydrazinecarbothioamide was synthesized *in situ* by mixing equimolar methanolic solutions of phenyl(pyridin-2-yl)methanone (0.0916 g, 0.5 mmol), hydrazinecarbothioamide (0.0455 g, 0.5 mmol) and 5 drops of glacial acetic acid for 2 h. The title complex was prepared by adapting a reported procedure (Philip *et al.*, 2006) by refluxing the above ligand solution and CdCl_2_·2.5H_2_O (0.1141 g, 0.5 mmol) for 3 h. The resulting solution was cooled at room temperature. Upon slow evaporation, yellow coloured product formed were collected, washed with few drops of methanol and dried over P_4_O_10_*in vacuo*. Yellow blocked shaped single crystals of the title compound suitable for X-ray analysis were obtained by recrystallization from methanol. The compound was obtained in 56%, yield (0.1320 g).

IR (KBr, \v in cm^-1^): 3439, 3225, 3125, 1603, 1380, 1314, 1213, 1147, 780, 659, 560.

### S3. Refinement

All H atoms on C were placed in calculated positions, guided by difference maps, with C—H bond distances of 0.93–0.96 Å. H atoms were assigned *U*_iso_(H) values of 1.2U_eq_(1.5 for methyl group). H3', H1', H4A and H4B were located from a difference Fourier map and refined isotropically. Omitted owing to bad disagreement were reflections (0 1 1), (0 0 2) and (0 2 0).

### Crystal data

**Table:**

[CdCl_2_(C_13_H_12_N_4_S)]·CH_4_O	*F*(000) = 936
*M**_r_* = 471.67	*D*_x_ = 1.713 Mg m^−^^3^
Monoclinic, *P*2_1_/*n*	Mo *K*α radiation, λ = 0.71073 Å
*a* = 7.7213 (4) Å	Cell parameters from 8955 reflections
*b* = 12.9759 (8) Å	θ = 2.7–28.1°
*c* = 18.3318 (11) Å	µ = 1.61 mm^−^^1^
β = 95.248 (2)°	*T* = 293 K
*V* = 1828.98 (18) Å^3^	Block, yellow
*Z* = 4	0.30 × 0.25 × 0.20 mm

### Data collection

**Table:**

Bruker Kappa APEXII CCD diffractometer	4392 independent reflections
Radiation source: fine-focus sealed tube	3804 reflections with *I* > 2σ(*I*)
Detector resolution: 8.33 pixels mm^-1^	*R*_int_ = 0.031
ω and φ scan	θ_max_ = 28.0°, θ_min_ = 2.7°
Absorption correction: multi-scan (*SADABS*; Bruker, 2004)	*h* = −9→10
*T*_min_ = 0.624, *T*_max_ = 0.725	*k* = −17→13
13483 measured reflections	*l* = −24→24

### Refinement

**Table:**

Refinement on *F*^2^	Hydrogen site location: inferred from neighbouring sites
Least-squares matrix: full	H atoms treated by a mixture of independent and constrained refinement
*R*[*F*^2^ > 2σ(*F*^2^)] = 0.028	*w* = 1/[σ^2^(*F*_o_^2^) + (0.0364*P*)^2^ + 1.2328*P*] where *P* = (*F*_o_^2^ + 2*F*_c_^2^)/3
*wR*(*F*^2^) = 0.074	(Δ/σ)_max_ = 0.001
*S* = 0.99	Δρ_max_ = 0.50 e Å^−^^3^
4392 reflections	Δρ_min_ = −0.60 e Å^−^^3^
226 parameters	Extinction correction: *SHELXL*, Fc^*^=kFc[1+0.001xFc^2^λ^3^/sin(2θ)]^-1/4^
5 restraints	Extinction coefficient: 0.0143 (5)

### Special details

**Table:**

Geometry. All e.s.d.'s (except the e.s.d. in the dihedral angle between two l.s. planes) are estimated using the full covariance matrix. The cell e.s.d.'s are taken into account individually in the estimation of e.s.d.'s in distances, angles and torsion angles; correlations between e.s.d.'s in cell parameters are only used when they are defined by crystal symmetry. An approximate (isotropic) treatment of cell e.s.d.'s is used for estimating e.s.d.'s involving l.s. planes.

### Fractional atomic coordinates and isotropic or equivalent isotropic displacement parameters (Å^2^)

**Table:**

	*x*	*y*	*z*	*U*_iso_*/*U*_eq_	
C1	0.6778 (3)	−0.0088 (2)	0.60386 (14)	0.0421 (6)	
H1	0.6464	0.0012	0.5542	0.050*	
C1S	1.5904 (6)	0.1528 (4)	0.8959 (2)	0.0876 (14)	
H1S1	1.7000	0.1843	0.9116	0.131*	
H1S2	1.6102	0.0858	0.8762	0.131*	
H1S3	1.5223	0.1463	0.9369	0.131*	
C2	0.5687 (3)	−0.0651 (2)	0.64401 (17)	0.0507 (7)	
H2	0.4663	−0.0931	0.6218	0.061*	
C3	0.6140 (4)	−0.0790 (3)	0.71739 (17)	0.0526 (7)	
H3	0.5415	−0.1157	0.7458	0.063*	
C4	0.7687 (3)	−0.0379 (2)	0.74891 (15)	0.0416 (6)	
H4	0.8021	−0.0473	0.7985	0.050*	
C5	0.8727 (3)	0.01721 (18)	0.70534 (12)	0.0314 (4)	
C6	1.0420 (3)	0.06173 (18)	0.73536 (12)	0.0306 (4)	
C7	1.0948 (3)	0.05746 (19)	0.81515 (12)	0.0330 (5)	
C8	1.0602 (4)	0.1385 (2)	0.85957 (16)	0.0522 (7)	
H8	1.0030	0.1965	0.8397	0.063*	
C9	1.1105 (5)	0.1341 (3)	0.93427 (17)	0.0629 (9)	
H9	1.0881	0.1895	0.9642	0.075*	
C10	1.1920 (5)	0.0491 (3)	0.96346 (16)	0.0654 (9)	
H10	1.2226	0.0455	1.0137	0.078*	
C11	1.2297 (5)	−0.0314 (3)	0.91957 (18)	0.0739 (11)	
H11	1.2879	−0.0889	0.9399	0.089*	
C12	1.1816 (4)	−0.0279 (2)	0.84519 (16)	0.0557 (7)	
H12	1.2075	−0.0827	0.8154	0.067*	
C13	1.3920 (3)	0.18172 (19)	0.65912 (12)	0.0330 (5)	
N1	0.8267 (2)	0.03206 (16)	0.63337 (10)	0.0334 (4)	
N2	1.1338 (2)	0.10222 (16)	0.68830 (10)	0.0319 (4)	
N3	1.2894 (3)	0.14580 (17)	0.71024 (11)	0.0361 (5)	
S1	1.33814 (8)	0.17757 (6)	0.56772 (3)	0.03929 (15)	
Cl1	0.87665 (9)	0.10250 (6)	0.44174 (3)	0.04381 (16)	
Cl2	0.88268 (9)	0.29835 (6)	0.61058 (4)	0.04826 (16)	
Cd1	1.00948 (2)	0.13266 (2)	0.56724 (2)	0.03757 (8)	
O1S	1.5008 (3)	0.2144 (2)	0.84178 (12)	0.0690 (7)	
N4	1.5417 (3)	0.2202 (2)	0.68671 (12)	0.0442 (5)	
H3'	1.326 (4)	0.147 (2)	0.7569 (7)	0.051 (9)*	
H1'	1.469 (6)	0.2722 (18)	0.857 (2)	0.094 (15)*	
H4A	1.572 (3)	0.227 (2)	0.7320 (6)	0.041 (8)*	
H4B	1.618 (3)	0.244 (2)	0.6613 (12)	0.049 (8)*	

### Atomic displacement parameters (Å^2^)

**Table:**

	*U*^11^	*U*^22^	*U*^33^	*U*^12^	*U*^13^	*U*^23^
C1	0.0354 (12)	0.0465 (15)	0.0425 (13)	−0.0005 (11)	−0.0063 (10)	−0.0023 (11)
C1S	0.072 (3)	0.121 (4)	0.068 (2)	0.026 (2)	−0.006 (2)	−0.008 (2)
C2	0.0336 (13)	0.0547 (17)	0.0611 (17)	−0.0111 (12)	−0.0094 (11)	0.0044 (14)
C3	0.0382 (14)	0.0572 (18)	0.0623 (17)	−0.0142 (12)	0.0041 (12)	0.0157 (15)
C4	0.0363 (12)	0.0457 (14)	0.0422 (12)	−0.0057 (10)	0.0008 (10)	0.0085 (11)
C5	0.0290 (10)	0.0334 (12)	0.0317 (10)	−0.0004 (9)	0.0019 (8)	−0.0002 (9)
C6	0.0304 (10)	0.0323 (11)	0.0290 (10)	−0.0006 (9)	0.0018 (8)	0.0000 (9)
C7	0.0327 (11)	0.0383 (13)	0.0280 (10)	−0.0060 (9)	0.0018 (8)	0.0016 (9)
C8	0.0618 (18)	0.0534 (18)	0.0414 (14)	0.0111 (13)	0.0053 (13)	−0.0063 (12)
C9	0.077 (2)	0.074 (2)	0.0386 (15)	−0.0028 (17)	0.0085 (14)	−0.0200 (15)
C10	0.084 (2)	0.079 (2)	0.0308 (13)	−0.0169 (19)	−0.0070 (14)	0.0060 (15)
C11	0.109 (3)	0.062 (2)	0.0453 (17)	0.004 (2)	−0.0225 (18)	0.0132 (15)
C12	0.079 (2)	0.0440 (16)	0.0415 (14)	0.0086 (15)	−0.0104 (13)	−0.0027 (12)
C13	0.0301 (11)	0.0344 (12)	0.0350 (11)	−0.0020 (9)	0.0055 (9)	−0.0026 (9)
N1	0.0307 (9)	0.0377 (11)	0.0312 (9)	−0.0007 (8)	−0.0005 (7)	−0.0020 (8)
N2	0.0271 (9)	0.0381 (10)	0.0299 (9)	−0.0059 (8)	−0.0004 (7)	−0.0006 (8)
N3	0.0295 (10)	0.0521 (13)	0.0261 (9)	−0.0099 (8)	−0.0006 (7)	−0.0007 (8)
S1	0.0343 (3)	0.0535 (4)	0.0306 (3)	−0.0059 (3)	0.0057 (2)	0.0016 (3)
Cl1	0.0462 (3)	0.0522 (4)	0.0309 (3)	0.0028 (3)	−0.0080 (2)	−0.0044 (3)
Cl2	0.0479 (4)	0.0491 (4)	0.0494 (3)	0.0008 (3)	0.0134 (3)	−0.0027 (3)
Cd1	0.03411 (11)	0.05315 (14)	0.02497 (10)	−0.00521 (7)	0.00006 (6)	0.00039 (7)
O1S	0.0751 (15)	0.091 (2)	0.0399 (11)	0.0034 (14)	0.0001 (10)	−0.0176 (12)
N4	0.0326 (11)	0.0592 (15)	0.0409 (11)	−0.0146 (10)	0.0046 (9)	−0.0074 (11)

### Geometric parameters (Å, º)

**Table:**

C1—N1	1.334 (3)	C9—C10	1.356 (5)
C1—C2	1.378 (4)	C9—H9	0.9300
C1—H1	0.9300	C10—C11	1.366 (5)
C1S—O1S	1.405 (5)	C10—H10	0.9300
C1S—H1S1	0.9600	C11—C12	1.381 (4)
C1S—H1S2	0.9600	C11—H11	0.9300
C1S—H1S3	0.9600	C12—H12	0.9300
C2—C3	1.370 (4)	C13—N4	1.317 (3)
C2—H2	0.9300	C13—N3	1.363 (3)
C3—C4	1.385 (4)	C13—S1	1.690 (2)
C3—H3	0.9300	N1—Cd1	2.341 (2)
C4—C5	1.383 (3)	N2—N3	1.356 (3)
C4—H4	0.9300	N2—Cd1	2.3690 (19)
C5—N1	1.349 (3)	N3—H3'	0.876 (10)
C5—C6	1.488 (3)	S1—Cd1	2.6030 (6)
C6—N2	1.278 (3)	Cl1—Cd1	2.4630 (6)
C6—C7	1.483 (3)	Cl2—Cd1	2.5208 (7)
C7—C8	1.371 (4)	O1S—H1'	0.846 (10)
C7—C12	1.382 (4)	N4—H4A	0.845 (9)
C8—C9	1.390 (4)	N4—H4B	0.841 (10)
C8—H8	0.9300		
			
N1—C1—C2	122.7 (2)	C11—C10—H10	119.8
N1—C1—H1	118.7	C10—C11—C12	120.2 (3)
C2—C1—H1	118.7	C10—C11—H11	119.9
O1S—C1S—H1S1	109.5	C12—C11—H11	119.9
O1S—C1S—H1S2	109.5	C11—C12—C7	119.8 (3)
H1S1—C1S—H1S2	109.5	C11—C12—H12	120.1
O1S—C1S—H1S3	109.5	C7—C12—H12	120.1
H1S1—C1S—H1S3	109.5	N4—C13—N3	114.2 (2)
H1S2—C1S—H1S3	109.5	N4—C13—S1	121.38 (18)
C3—C2—C1	118.7 (2)	N3—C13—S1	124.39 (17)
C3—C2—H2	120.7	C1—N1—C5	118.7 (2)
C1—C2—H2	120.7	C1—N1—Cd1	123.32 (17)
C2—C3—C4	119.5 (3)	C5—N1—Cd1	117.97 (14)
C2—C3—H3	120.3	C6—N2—N3	120.16 (19)
C4—C3—H3	120.3	C6—N2—Cd1	119.91 (15)
C5—C4—C3	118.8 (2)	N3—N2—Cd1	118.72 (14)
C5—C4—H4	120.6	N2—N3—C13	119.59 (19)
C3—C4—H4	120.6	N2—N3—H3'	120 (2)
N1—C5—C4	121.6 (2)	C13—N3—H3'	121 (2)
N1—C5—C6	116.77 (19)	C13—S1—Cd1	99.32 (8)
C4—C5—C6	121.6 (2)	N1—Cd1—N2	68.51 (6)
N2—C6—C7	124.2 (2)	N1—Cd1—Cl1	100.12 (5)
N2—C6—C5	115.62 (19)	N2—Cd1—Cl1	161.25 (6)
C7—C6—C5	120.20 (19)	N1—Cd1—Cl2	92.43 (5)
C8—C7—C12	119.4 (2)	N2—Cd1—Cl2	89.01 (5)
C8—C7—C6	120.4 (2)	Cl1—Cd1—Cl2	106.82 (2)
C12—C7—C6	120.2 (2)	N1—Cd1—S1	139.20 (5)
C7—C8—C9	120.1 (3)	N2—Cd1—S1	73.91 (5)
C7—C8—H8	120.0	Cl1—Cd1—S1	111.18 (2)
C9—C8—H8	120.0	Cl2—Cd1—S1	102.39 (2)
C10—C9—C8	120.0 (3)	C1S—O1S—H1'	114 (3)
C10—C9—H9	120.0	C13—N4—H4A	124.5 (17)
C8—C9—H9	120.0	C13—N4—H4B	124.1 (18)
C9—C10—C11	120.4 (3)	H4A—N4—H4B	111 (2)
C9—C10—H10	119.8		
			
N1—C1—C2—C3	0.6 (5)	C8—C7—C12—C11	1.2 (5)
C1—C2—C3—C4	−1.1 (5)	C6—C7—C12—C11	−179.7 (3)
C2—C3—C4—C5	0.7 (5)	C2—C1—N1—C5	0.3 (4)
C3—C4—C5—N1	0.2 (4)	C2—C1—N1—Cd1	−177.9 (2)
C3—C4—C5—C6	−178.9 (3)	C4—C5—N1—C1	−0.7 (4)
N1—C5—C6—N2	−5.7 (3)	C6—C5—N1—C1	178.4 (2)
C4—C5—C6—N2	173.4 (2)	C4—C5—N1—Cd1	177.54 (19)
N1—C5—C6—C7	174.3 (2)	C6—C5—N1—Cd1	−3.3 (3)
C4—C5—C6—C7	−6.6 (4)	C7—C6—N2—N3	−0.6 (4)
N2—C6—C7—C8	86.1 (3)	C5—C6—N2—N3	179.3 (2)
C5—C6—C7—C8	−93.9 (3)	C7—C6—N2—Cd1	−167.91 (17)
N2—C6—C7—C12	−93.0 (3)	C5—C6—N2—Cd1	12.1 (3)
C5—C6—C7—C12	87.0 (3)	C6—N2—N3—C13	175.0 (2)
C12—C7—C8—C9	−0.7 (5)	Cd1—N2—N3—C13	−17.5 (3)
C6—C7—C8—C9	−179.8 (3)	N4—C13—N3—N2	−178.5 (2)
C7—C8—C9—C10	−0.8 (5)	S1—C13—N3—N2	0.8 (3)
C8—C9—C10—C11	1.8 (6)	N4—C13—S1—Cd1	−167.7 (2)
C9—C10—C11—C12	−1.4 (6)	N3—C13—S1—Cd1	13.1 (2)
C10—C11—C12—C7	−0.1 (6)		

### Hydrogen-bond geometry (Å, º)

**Table:**

*D*—H···*A*	*D*—H	H···*A*	*D*···*A*	*D*—H···*A*
N4—H4*A*···O1*S*	0.85 (1)	2.14 (2)	2.890 (3)	148 (2)
N4—H4*B*···Cl2^i^	0.84 (1)	2.43 (1)	3.253 (2)	167 (3)
N3—H3′···O1*S*	0.88 (1)	2.15 (2)	2.924 (3)	147 (3)
O1*S*—H1′···Cl1^ii^	0.85 (1)	2.40 (2)	3.201 (3)	158 (4)
C2—H2···Cl1^iii^	0.93	2.80	3.680 (3)	159

Symmetry codes: (i) *x*+1, *y*, *z*; (ii) *x*+1/2, −*y*+1/2, *z*+1/2; (iii) −*x*+1, −*y*, −*z*+1.
